# Impact of Pharmacist Involvement on Telehealth Transitional Care Management (TCM) for High Medication Risk Patients

**DOI:** 10.3390/pharmacy7040158

**Published:** 2019-11-25

**Authors:** Jessica Cole, Nick Wilkins, Maeghan Moss, Danny Fu, Paige Carson, Linda Xiong

**Affiliations:** Atrium Health Cabarrus Medication Management Clinic, 315 Medical Park Drive NE, Suite 204, Concord, NC 28025, USA; Nick.Wilkins@atriumhealth.org (N.W.); Maeghan.Moss@atriumhealth.org (M.M.); Danny.Fu@atriumhealth.org (D.F.); Paige.Carson@atriumhealth.org (P.C.); li.xiong@wingate.edu (L.X.)

**Keywords:** transitional care management, transitions of care, care transitions, medication access, medication reconciliation, medication management, polypharmacy, hospital readmissions

## Abstract

This pilot study sought to evaluate the impact of pharmacist involvement in the preexisting telehealth transitional care management (TCM) program at Atrium Health on the quality and safety of the medication discharge process for high medication risk patients. Eligible participants were those 18 years of age or older with moderate-to-high risk for hospital readmission who were contacted by a TCM Nurse, identified as high medication risk patients, and referred to the TCM Pharmacist from September 2018 through February 2019. The TCM Pharmacist contacted patients by phone, completed a comprehensive medication review, identified medication list discrepancies (MLDs) and medication-related problems (MRPs), and made interventions or recommendations to primary care providers. Primary endpoints included the number and types of MLDs identified, number and types of MRPs identified, and the rate of unplanned 30-day hospital readmissions. Seventy-six patients were enrolled, and 78 MLDs and 108 MRPs were identified. Of the identified MRPs, 74.1% were resolved. A relative risk reduction of 36.8% was achieved for 30-day hospital readmissions for those with high medication risk contacted by the TCM Pharmacist compared to those only contacted by the TCM Nurse. Overall, TCM Pharmacists identified and resolved 80 medication-related problems, improved access to medication therapy, provided comprehensive medication counseling, and bridged gaps in care following hospital discharge.

## 1. Introduction

The cost of healthcare services in the United States is disproportionately concentrated among a small percentage of patients with complex health needs [1]. This patient population typically includes older adults who possess multiple chronic conditions and/or functional limitations [2]. Targeted care models addressing this risk-stratified patient population can achieve improvement in patient outcomes and reduce healthcare spending through the utilization of interdisciplinary teamwork, fluid care transitions, and a standardized medication management process [2].

Transitional care management (TCM) involves services provided to a patient with complex health needs following discharge from the facility setting [3]. Achieving effective transitions between healthcare settings is integral in preventing hospital readmissions and ensuring patient safety. Hospital readmission rates within 30 days have been reported to be as high as 20% for Medicare patients [4]. The rate of hospital readmissions within 30 days has become a standard quality measure, and the Centers for Medicare and Medicaid Services enact monetary penalties for 30-day hospital readmissions in select high-risk disease states [5]. Some common factors impeding effective transitions between care settings include insufficient patient or caregiver education, a lack of communication between inpatient and ambulatory care providers, and inadequate assessment of medication access and health literacy prior to discharge [6]. Medication-related problems (MRPs) such as duplications of drug therapy, inadequate drug monitoring, and unintentional discontinuation of medications during the hospital admission process may occur when transitioning from inpatient to outpatient care, as nearly two thirds of post-discharge adverse events are medication-related [6,7]. 

Project Re-Engineered Discharge, also referred to as Project RED, determined that patients who met with a nurse discharge advocate prior to discharge and were contacted by phone by a pharmacist 2–4 days post-discharge had a 30% lower readmission rate within 30 days and a better understanding of their medications than those who did not receive this service [8]. Similarly, Dempsey et al. identified 244 recommendations for therapeutic optimization and found a 20% reduction in the 30-day hospital readmission rate for patients with heart failure who met with a transitions-of-care pharmacist before their outpatient cardiovascular clinic appointment or during the hospitalization [6]. Additionally, Cavanaugh et al. determined that follow-up with an interdisciplinary team coordinated by a Clinical Pharmacist Practitioner decreased 30-day hospital readmission rates compared with those who had follow-up visits with a physician-only team [9]. Finally, Odeh et al. implemented a pharmacist-led telephonic intervention following hospital discharge and found a 9.9% reduction in 30-day readmission rates and a 15.2% reduction in 90-day readmission rates compared to patients who did not receive the service [10]. As medication experts, pharmacists can play a vital role in identifying medication-related problems, providing medication counseling, ensuring medication access, and facilitating appropriate outpatient follow-up after hospital discharge in order to improve patient care.

Currently, TCM Nurses at Atrium Health contact moderate-to-high risk discharge patients by phone to coordinate post-discharge medical appointments and address patient questions. This study sought to develop a TCM Pharmacist position and evaluate the impact of pharmacist involvement in the telehealth TCM program on the quality and safety of the medication discharge process for high medication risk patients.

## 2. Materials and Methods

### 2.1. Research Design

The study was designed as a prospective, investigator-initiated pilot study affiliated with Atrium Health Cabarrus. Atrium Health is a large healthcare network with more than 40 hospitals and 900 care locations serving patients in Georgia, North Carolina, and South Carolina. Atrium Health Cabarrus is a 457-bed, not-for-profit medical center located in Concord, North Carolina, United States. The Institutional Review Board deemed this study a quality improvement project on 24 August 2018. A postgraduate year-1 pharmacy resident and a team of clinical pharmacists practicing in the ambulatory care setting at Atrium Health Cabarrus Medication Management Clinic served as the TCM Pharmacist and conducted the telehealth intervention. A fourth-year student pharmacist monitored by a TCM Pharmacist assisted with the intervention and data collection.

### 2.2. Methodology

Eligible participants were those 18 years of age and older with moderate-to-high risk for hospital readmission who were contacted by a TCM Nurse after hospital discharge from Atrium Health Cabarrus, identified as high medication risk patients, and referred to a TCM Pharmacist from September 2018 through February 2019. Atrium Health’s electronic health record (EHR) calculates a readmission risk score based off of the following data: age; body mass index (BMI); insurance; acuity; history of hospital discharge against-medical-advice (AMA); prior skilled nursing facility admission; emergency department visit, inpatient admission, or observation stay within the last six months; comorbidity index; polypharmacy; and high risk medication use. The populated score equates to low (39 or less), moderate (40 to 59), or high (60 or more) risk for hospital readmission. Patients classified as moderate or high risk for readmission were automatically added to the TCM Nurse work queue.

**Figure 1:**
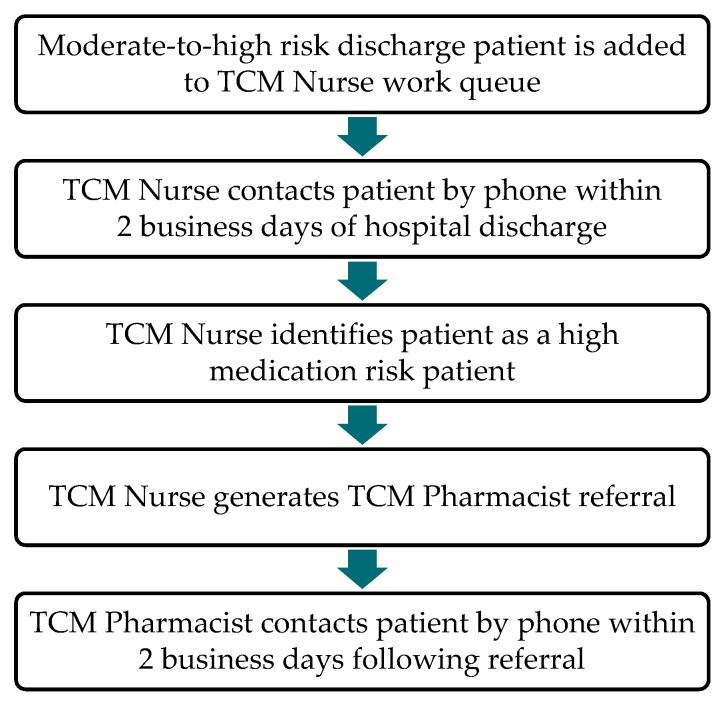
Transitional care management (TCM) Pharmacist referral process.

Following the preexisting workflow, the TCM Nurse contacted patients by phone within 2 business days of hospital discharge to complete a questionnaire which evaluated the patient/caregiver’s knowledge of the patient’s condition and treatment. The TCM Nurse also discussed warning signs/symptoms that require follow-up and reviewed the discharge instructions and discharge medication list. If the patient was admitted due to chronic obstructive pulmonary disease (COPD), diabetes mellitus, heart failure, or an acute myocardial infarction (MI), the TCM Nurse asked a preset list of disease state-specific questions. During the TCM Pharmacist pilot, the TCM Nurse could refer the patient to a TCM Pharmacist if the patient was identified as having high medication risk. High medication risk patients were defined as those with polypharmacy (defined as 9 or more medications), medication nonadherence (as identified during the TCM Nurse questionnaire), low medication literacy (as determined during the discharge medication review), high risk medications (including insulin, anticoagulants, and antiplatelet medications), high risk admission diagnoses (including acute MI, heart failure, pneumonia, COPD, coronary artery bypass grafting (CABG), total hip or total knee arthroplasty, and uncontrolled diabetes), and/or with specific clinical scenarios at the discretion of the TCM Nurse. The TCM Pharmacist referral process is outlined in Figure 1.

The referral was made electronically within the EHR. The TCM Pharmacist monitored the referral box on weekdays. When a new patient case was received, the TCM Pharmacist reviewed the reason for referral and documentation from the recent hospitalization. The TCM Pharmacist completed a chart review and began a comprehensive medication review by assessing the patient’s renal and hepatic function; potential drug–drug interactions, drug–disease interactions, and side effects; indications for therapy; disease control; and insurance coverage. The TCM Pharmacist then contacted the patient by phone within 2 business days of receiving the referral to address any medication-related concerns and reconcile the medication list. The TCM Pharmacist completed the comprehensive medication review during the telephone encounter and identified any medication list discrepancies (MLDs) and/or MRPs. The TCM Pharmacist enacted interventions if appropriate; contacted a pharmacy, insurance company, or patient assistance program; and/or made recommendations to the patient’s primary care provider via the EHR messaging system or telephonic interactions.

The TCM Pharmacist documented interventions and time spent on encounters in the PharmD Clinical Interventions Documentation Ad Hoc form and in the patient’s EHR. The PharmD Interventions Documentation Ad Hoc form is a reporting tool for MRPs created within the EHR following parameters defined by Crisp and colleagues [11]. A follow-up phone call was completed by a TCM Pharmacist within 14–21 days after the initial call to ensure MRPs were resolved and no further medication-related concerns persisted. If warranted, further documentation was completed in the PharmD Clinical Interventions Documentation Ad Hoc form and the patient’s EHR.

### 2.3. Primary and Secondary Endpoints

Primary endpoints included the number and types of MLDs identified, the number and types of MRPs identified, and the number and rate of unplanned 30-day hospital readmissions. Secondary endpoints included the number of MRPs resolved, number of recommendations accepted by providers, number of calls attempted, and number of calls completed. Time to first contact and time spent on the encounter were also analyzed.

### 2.4. Data Analysis

A sample size of 95 pairs was determined to detect a 10% reduction in the 30-day hospital readmission rate between the intervention and control groups and achieve 80% power with an alpha level of 0.05. Data were collected from the PharmD Clinical Interventions Documentation Ad Hoc form and the patient’s EHR. Data were stored and analyzed using the Research Electronic Data Capture (REDCap) database without patient identifiers. Endpoints were analyzed using descriptive statistics, including counts and percentages. The primary analysis of the 30-day hospital readmission rate endpoint comparing patients contacted by the TCM Pharmacist and patients who declined services or could not be reached was evaluated using a two-sided Barnard test. A two-tailed p-value of less than 0.05 was considered statistically significant.

## 3. Results

### 3.1. Patient Enrollment

**Figure 2:**
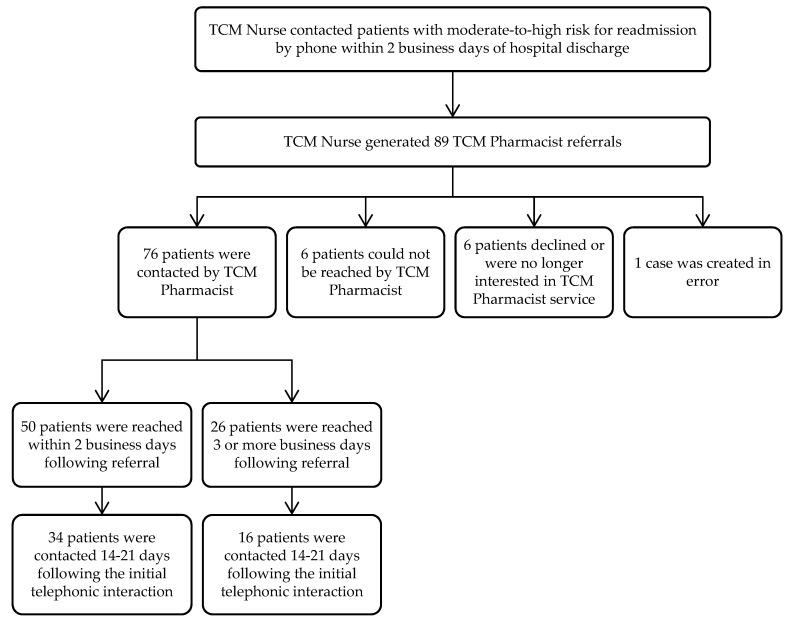
TCM Pharmacist patient enrollment.

Eighty-nine TCM Pharmacist referrals were obtained from September 2018 through February 2019. Seventy-six patients were contacted by the TCM Pharmacist, six patients were not reached, six patients declined or were no longer interested, and one case was created in error, as depicted in Figure 2.

The average time to initial contact by the TCM Pharmacist was 2.1 business days following referral. Of the 76 patients contacted by the TCM Pharmacist, 50 were reached within 2 business days of referral from the TCM Nurse. An additional 8 patients were contacted within 3–4 business days following referral. Fifteen additional patients were contacted within 10 business days following referral. Three patients were not reached until 12 or more business days following referral. The range for time to initial contact was 0 to 24 business days following referral.

#### 3.1.1. Baseline Characteristics

The average age of patients contacted by the TCM Pharmacist was 65 years old. More than half were female (59.2%) and Caucasian (77.9%). The three most common comorbidities were hypertension (73.7%), dyslipidemia (52.6%), and type 2 diabetes mellitus (50.0%). Thirty-two patients (42.1%) were taking a narcotic or sedative, 30 patients (39.5%) were on anticoagulant therapy, and 24 patients (31.6%) were using insulin therapy. On average, the patients had nine chronic conditions and 15 active medications at the time of hospital discharge. The patient population’s baseline demographics are summarized below in Table 1. Table 1 also includes the baseline characteristics for the 12 individuals in the Non-TCM Pharmacist Intervention Group who were referred to the TCM Pharmacist but were not reached or declined the service.

#### 3.1.2. Reason for TCM Pharmacist Referral

**Figure 3:**
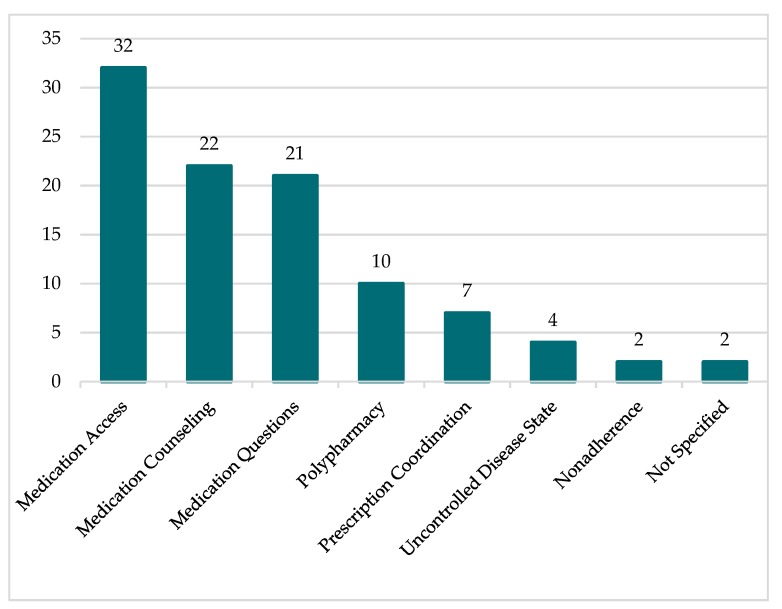
Reason for referral to TCM Pharmacist.

The most common reasons for TCM Pharmacist referral were medication access, medication counseling, and medication questions, followed by polypharmacy, prescription coordination, uncontrolled disease state, and nonadherence. Two cases did not include a reason for TCM Pharmacist referral. Eleven referrals included multiple reasons for referral. The specific frequency of each referral type is depicted in Figure 3.

### 3.2. Medication List Discrepancies (MLDs)

Seventy-six MLDs were identified through TCM Pharmacist involvement. Forty-four MLDs were categorized as Addition Errors, where a medication was included on the medication list which should not have been. Eighteen Omission Errors, where the medication was not on the list but should have been, were found. Furthermore, 16 Details Errors, where the medication on the list had incorrect or missing information, were identified.

### 3.3. Medication-Related Problems (MRPs)

The TCM Pharmacist identified 108 MRPs. A majority were categorized as nonadherence or medication access issues (54.6%), with 27 of these MRPs being due to medication cost. Sixteen MRPs were related to suboptimal dosing, duration, frequency, or administration, while 13 MRPs fell under the category of suboptimal drug. Table 2 depicts the categorization of the 108 MRPs into broad groups and more specific subtypes.

Of the 108 MRPs identified, 49 MRPs were resolved directly by the TCM Pharmacist through medication counseling, patient education, referral to financial resources, and/or by practicing under North Carolina’s Clinical Pharmacist Practitioner (CPP) act [12]. Forty-seven recommendations were made to primary care providers, with 31 recommendations being accepted and implemented. Of the remaining 28 MRPs which were not resolved, 16 were due to the primary care provider not accepting the recommendation and 12 were due to the patient not accepting or implementing the TCM Pharmacist intervention. Overall, 80 MRPs, or 74.1%, of the MRPs identified were resolved.

### 3.4. Unplanned 30-Day Hospital Readmission Rate

Of the 76 patients contacted by the TCM Pharmacist, 12 patients were readmitted for an unplanned hospitalization and one patient was readmitted for a planned procedure at an Atrium Health facility within 30 days of hospital discharge. Five patients were seen under observation, and eight patients visited the emergency department at an Atrium Health facility within the 30-day period following hospital discharge.

Of the six patients who were not reached and the six patients who declined TCM Pharmacist involvement, three patients were re-hospitalized for an unplanned hospitalization within 30 days of hospital discharge at an Atrium Health facility. Table 3 depicts the readmission rates for the two groups and demonstrates a relative risk reduction of 36.8% for those contacted by the TCM Pharmacist service compared to those who were not reached or declined the TCM Pharmacist service.

### 3.5. Additional Secondary Endpoints

The TCM Pharmacist attempted 249 calls to the 89 patients referred to the service. Eighty-five initial or action calls were completed to the 76 patients contacted. Fifty-six follow-up calls were completed to assess the resolution of MRPs.

For the 76 patients reached by the TCM Pharmacist, 3712 min were spent on the TCM Pharmacist encounter, including the time spent conducting a chart review, contacting the patient, and intervening on identified MRPs and MLDs. The average TCM Pharmacist encounter time was approximately 49 min. The median amount of time spent on a TCM Pharmacist encounter was 42 min.

## 4. Discussion

As evident from the baseline characteristics, the patient population referred to the TCM Pharmacist service consisted of patients with multiple chronic conditions and complex medication regimens. TCM Pharmacists identified over 100 MRPs affecting the quality and safety of medication therapy following hospital discharge for 76 patients with moderate or high risk for hospital readmission and high medication risk. TCM Pharmacists independently resolved a majority of the MRPs identified through comprehensive medication counseling, patient/caregiver education, referral to financial resources, and/or by practicing under the CPP act. Through these interventions, TCM Pharmacists improved access to medication therapy, clarified complex medication regimens, answered medication- and disease-related questions, and addressed nonadherence post-discharge through telehealth services.

Within Surbhi et al., the frequency and type of medication therapy problems and discrepancies were investigated for high healthcare utilizers [13]. The estimated cost avoidance per each drug therapy problem identified was also evaluated and determined to be $293.30 [13]. As such, the 108 MRPs identified by TCM Pharmacists during the pilot study equates to an estimated cost avoidance of $31,676.40. Ultimately, utilization of the TCM Pharmacist position allowed for medication optimization post-discharge and potential cost avoidance.

Although statistical significance between those contacted by the TCM Pharmacist and those who declined services or could not be reached was not achieved, the 30-day readmission rate of 15.8% for patients contacted by the TCM Pharmacist was lower than the readmission rate of 17.8% reported for Medicare beneficiaries in 2012 [14]. As previously highlighted, a majority of the patient population contacted by the TCM Pharmacist consisted of Medicare beneficiaries. As such, pharmacist involvement in TCM services could lead to reductions in 30-day readmission rates, although further analysis is required.

The study should be interpreted while considering the following limitations. Firstly, the pilot study had a small sample size and did not reach the enrollment necessary to reach 80% power. Additionally, a comparable control group of adequate size was not identified and limited any potential for statistical analysis between the control and treatment group. Furthermore, the baseline characteristics between the control and treatment group were not balanced, and additional analysis to control for confounding variables is necessary. Due to limited TCM Pharmacist availability, only TCM Nurses serving Atrium Health Cabarrus were able to refer patients to the TCM Pharmacist service. Also, the referrals were dependent on the TCM Nurses identifying and referring patients who would benefit from TCM Pharmacist intervention. Lastly, the readmission data was limited, as only access to Atrium Health facility data was available. 

Moving forward, additional staff will be trained to serve as the TCM Pharmacist at Atrium Health Cabarrus Medication Management Clinic. If additional resources and pharmacist time is designated to the TCM Pharmacist service, pharmacist involvement in the TCM program will be expanded to cover additional hospitals within the Atrium Health system. Additionally, grant funding opportunities are currently being pursued to support the expansion and continuation of the TCM Pharmacist service.

## 5. Conclusions

Overall, pharmacist involvement in the telehealth transitional care management program improved the quality and safety of the medication discharge process for high medication risk patients through the identification and resolution of 80 medication-related problems.

## Figures and Tables

**Table 1:** Baseline characteristics of Transitional Care Management (TCM) Pharmacist patient population.

Variable	TCM Pharmacist Intervention Group	Non-TCM Pharmacist Intervention Group
	(n = 76)	(n = 12)
**Age, years**		
Mean (Range)	65 (25–88)	61 (38–77)
**Sex, n (%)**		
Female	45 (59.2)	8 (66.7)
Male	31 (40.8)	4 (33.3)
**Race, n (%)**		
African American	14 (18.2)	5 (41.7)
American Indian or Alaska Native	2 (2.6)	0 (0.0)
Asian	1 (1.3)	0 (0.0)
Caucasian	60 (77.9)	6 (50.0)
Unknown	0 (0.0)	1 (8.3)
**Insurance, n (%)**		
Commercial	10 (13.1)	3 (25.0)
Medicaid	11 (14.5)	0 (0.0)
Medicare	42 (55.3)	7 (58.3)
Medicare/Medicaid	7 (9.2)	1 (8.3)
Self-Pay	6 (7.9)	1(8.3)
**Medical History, n (%)**		
Anxiety	26 (34.2)	4 (33.3)
Atrial Fibrillation	21 (27.6)	2 (16.7)
Chronic Kidney Disease	13 (17.1)	5 (41.7)
Chronic Obstructive Pulmonary Disease	26 (34.2)	2 (16.7)
Congestive Heart Failure	24 (31.6)	4 (33.3)
Coronary Artery Disease	26 (34.2)	3 (25.0)
Depression	22 (28.9)	2 (16.7)
Dyslipidemia	40 (52.6)	9 (75.0)
Hypertension	56 (73.7)	8 (66.7)
Obesity	40 (52.6)	5 (41.7)
Type 2 Diabetes Mellitus	38 (50.0)	7 (58.3)
**High Risk Medication Use, n (%)**		
Anticoagulants	30 (39.5)	4 (33.3)
Anti-infectives	27 (35.5)	2 (16.7)
Chemotherapy	1 (1.3)	0 (0.0)
Insulin	24 (31.6)	3 (25.0)
Narcotics and Other Sedatives	32 (42.1)	3 (25.0)
**Number of Chronic Conditions, n ^1^**		
Mean	9.1	8.8
Range	1–22	3–16
**Number of Active Medications, n ^1^**		
Mean	15.1	12.8
Range	2–29	6–19
**ED Visit Within Past 12 Months, n (%) ^2^**	49 (64.5)	6 (50.0)
**Average ED Visits Within Past 12 Months, n (Range) ^2^**	1.8 (1–19)	1.3 (0–4)
**Hospitalization Within Past 12 Months, n (%) ^2^**	49 (64.5)	9 (75.0)
**Average Hospitalizations Within Past 12 Months, n (Range) ^2^**	1.6 (1–11)	1.1 (0–3)

^1^ Count obtained at time of hospital discharge. ^2^ Prior to hospitalization generating TCM Pharmacist referral.

**Table 2:** Medication-related problems (MRPs) identified by TCM Pharmacist (n = 108).

Category	Frequency
**Undertreatment**	
▪ Additional Therapy Required	3
▪ Untreated Medical Condition	5
**Suboptimal Dosing, Duration, Frequency, Administration**	
▪ Administration Not Ideal/Correct	3
▪ Doses Too Low	6
▪ Doses Too High	7
**Monitoring Needed**	
▪ Assess/Prevent Potential Adverse Drug Event	7
**Suboptimal Drug**	
▪ Generic Alternative Available	1
▪ Safer Alternative Available	1
▪ No Indication or Need for Therapy	2
▪ Not Effective/Not Ideal	2
▪ Potential for Drug Interaction	3
▪ Therapeutic Duplication	4
**Adverse Drug Event Present**	
▪ Moderate	1
▪ Severe	4
**Nonadherence/Medication Access Issues**	
▪ Fear of Adverse Events	1
▪ Disbelief in Drug Effectiveness/Indication	2
▪ Forgets/Too Busy/Not a Priority	2
▪ Regimen Too Complex	2
▪ Felt Worse/Minor Side Effects	3
▪ Other	3
▪ Patient Not Aware of Medication Changes	4
▪ Misunderstood Directions	15
▪ Too Expensive	27

**Table 3:** Unplanned 30-day hospital readmission rate ^1^ for patients initially referred to TCM Pharmacist service.

	Unplanned 30-Day Hospital Readmission (n)	Readmission Rate (%)	*p*-Value
Patients Contacted by TCM Pharmacist Service (n = 76)	12	15.8	0.529
Patients Not Reached/Declined TCM Pharmacist Service (n = 12)	3	25.0	

^1^ At an Atrium Health facility.
